# Ecological Meltdown in the Firth of Clyde, Scotland: Two Centuries of Change in a Coastal Marine Ecosystem

**DOI:** 10.1371/journal.pone.0011767

**Published:** 2010-07-29

**Authors:** Ruth H. Thurstan, Callum M. Roberts

**Affiliations:** Environment Department, University of York, York, United Kingdom; Northern Fisheries Centre, Australia

## Abstract

**Background:**

The Firth of Clyde is a large inlet of the sea that extends over 100 km into Scotland's west coast.

**Methods:**

We compiled detailed fisheries landings data for this area and combined them with historical accounts to build a picture of change due to fishing activity over the last 200 years.

**Findings:**

In the early 19^th^ century, prior to the onset of industrial fishing, the Firth of Clyde supported diverse and productive fisheries for species such as herring (*Clupea harengus*, Clupeidae), cod (*Gadus morhua*, Gadidae), haddock (*Melanogrammus aeglefinus*, Gadidae), turbot (*Psetta maxima*, Scophthalmidae) and flounder (*Platichthys flesus*, Pleuronectidae). The 19^th^ century saw increased demand for fish, which encouraged more indiscriminate methods of fishing such as bottom trawling. During the 1880s, fish landings began to decline, and upon the recommendation of local fishers and scientists, the Firth of Clyde was closed to large trawling vessels in 1889. This closure remained in place until 1962 when bottom trawling for Norway lobster (*Nephrops norvegicus*, Nephropidae) was approved in areas more than three nautical miles from the coast. During the 1960s and 1970s, landings of bottomfish increased as trawling intensified. The trawl closure within three nautical miles of the coast was repealed in 1984 under pressure from the industry. Thereafter, bottomfish landings went into terminal decline, with all species collapsing to zero or near zero landings by the early 21^st^ century. Herring fisheries collapsed in the 1970s as more efficient mid-water trawls and fish finders were introduced, while a fishery for mid-water saithe (*Pollachius virens*, Gadidae) underwent a boom and bust shortly after discovery in the late 1960s. The only commercial fisheries that remain today are for *Nephrops* and scallops (*Pecten maximus*, Pectinidae).

**Significance:**

The Firth of Clyde is a marine ecosystem nearing the endpoint of overfishing, a time when no species remain that are capable of sustaining commercial catches. The evidence suggests that trawl closures helped maintain productive fisheries through the mid-20^th^ century, and their reopening precipitated collapse of bottomfish stocks. We argue that continued intensive bottom trawling for *Nephrops* with fine mesh nets will prevent the recovery of other species. This once diverse and highly productive environment will only be restored if trawl closures or other protected areas are re-introduced. The Firth of Clyde represents at a small scale a process that is occurring ocean-wide today, and its experience serves as a warning to others.

## Introduction

Throughout the North Atlantic, many once productive fisheries have exhibited severe declines as fishing activities have intensified during the last two hundred years [1], [2]. To compensate for decreasing catches, fishers have shifted onto other species, gradually targeting species at lower trophic levels [3]. Improvements in technology also enable vessels to exploit fishing grounds further offshore and in deeper waters, often using gear that is invasive and damaging to seabed habitats [4]. It is now recognised that marine biodiversity is rapidly declining as communities and habitats are degraded through fishing [5], and the recent decline of global fish landings has begun to reflect this [6]. It is becoming increasingly important to understand how much humans have degraded ocean ecosystems in order to set appropriate management targets for their recovery.

**Figure 1:**
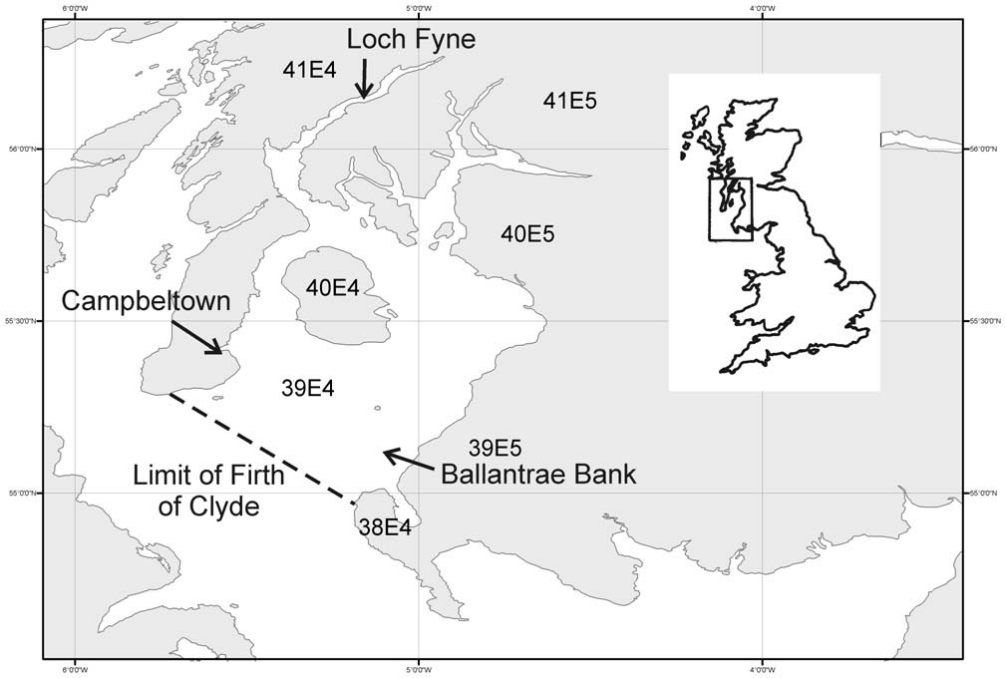
The Firth of Clyde. The dashed line indicates the limits of the Inner Firth of Clyde area. ICES statistical rectangles 39E4, 39E5, 40E4 and 40E5 indicate the Wider Firth of Clyde area. Ballantrae Banks was the site of a major herring fishery and spawning ground and has been exploited since the 16^th^ century, whilst Loch Fyne was famous for its high quality herring during the late 19^th^ century. Campbeltown was the main harbour for herring fleets of the 18^th^ century, but declined in importance as harbours were later constructed at other ports around the Firth of Clyde.

The Firth of Clyde is a large inlet of the sea that extends over 100 km into Scotland's west coast, and is the most southerly fjord in the North Atlantic (Figure 1). This environment has been subjected to a variety of human pressures over the centuries, such as harbour building and dredging, pollution and fishing. Throughout the last 200 years these impacts have intensified, leading to dramatic alterations within the Firth of Clyde marine environment.

During the 19^th^ century, diverse fisheries existed in the Firth of Clyde for herring (*Clupea harengus*, Clupeidae), cod (*Gadus morhua*, Gadidae), haddock (*Melanogrammus aeglefinus*, Gadidae), turbot (*Psetta maxima*, Scophthalmidae) and a variety of other species. Today these fisheries no longer exist; instead the main targets are invertebrates such as Norway lobster (*Nephrops norvegicus*, Nephropidae; hereafter called *Nephrops*) and scallops (*Pecten maximus*, Pectinidae). These are usually fished using otter trawls and dredges, both of which are dragged along the seabed, which disturbs bottom-living species and destroys fragile habitats [7]. This is a far cry from the less invasive static nets and hooks that predominated 130 years ago. Whilst it is likely the gear then in use also had some adverse effects upon fish populations, the industrialisation of fisheries from the 1880s has intensified exploitation beyond sustainable levels for many marine species.

In this paper we review the long-term impact of increasingly intensive and invasive fishing practises in the Firth of Clyde. We will show that overexploitation and destructive fishing activities such as trawling have transformed the Firth of Clyde, restructuring marine communities and reducing the abundance and diversity of commercially exploited populations. In this once highly productive environment, the endpoint of overfishing, a time when there are no commercially viable stocks left, is now in sight.

## Results

### Pre-industrial fisheries in the Firth of Clyde

It is clear from 18^th^ and 19^th^ century accounts that the Firth of Clyde harboured an abundance of species before the era of industrial fishing. The productive waters and shallow banks attracted shoals of herring which migrated to spawn and feed within the Firth, and these in turn attracted a diverse array of predators such as seabirds, whales, porpoises, and dogfish (*Squalus* spp., Squalidae) [8], [9] (see Appendix S1). The productive waters regularly attracted other visitors such as basking sharks (*Cetorhinus maximus*, Cetorhinidae), which were numerous enough to support local fisheries for their oil [10], [11].

This abundance of herring and other fish attracted fishers from outside the Firth of Clyde. As early as the 16^th^ century the Firth was visited on a seasonal basis by boats from the European continent following the herring shoals [12]. The local fleet expanded in the 18^th^ century. In 1750 it was estimated that up to 20,000 barrels of herring were caught by the Clyde boats [10], and in 1755 the value of herring exported from Campbeltown (a port on the outer Firth of Clyde, see Figure 1) alone was nearly £16,000, with a greater quantity exported from other ports within the Firth in the same year [13].

Large vessels from the east coast of Scotland travelled to the Firth of Clyde to exploit fisheries other than herring, such as flatfish (see Appendix S1). Skate were also regularly caught. The catch per unit effort for line fisheries could be very high, one account stating that a catch of 350 fish for 400 hooks was not an unusual occurrence [10]. Bottom-living species such as cod and ling (*Molva molva*, Gadidae) were important on a local level, but for centuries the most economically important fish was the herring. Areas such as the Ballantrae Bank (a shallow stretch of bank on the outer Firth of Clyde, see Figure 1) harboured suitable gravel substrate for the fish to spawn upon. High abundances of plankton throughout the Firth and the sea lochs created rich feeding grounds, forming the basis of a highly productive marine ecosystem [14].

By the 1870s the Ballantrae winter herring fishery was the most important in Scotland [9], with boats from as far away as the east coast coming to take part in the annual catch (see Table 1 for a timeline of major events). Fishers would target other species present, and mackerel (*Scomber scombrus*, Scombridae), haddock, whiting (*Merlangius merlangus*, Gadidae), turbot and flounder (*Platichthys flesus*, Pleuronectidae) fisheries also existed in the Clyde [15]. Species such as cod and haddock were targeted using longlines or handlines, and flatfish such as turbot were caught using static nets to be exported as prime fish to large urban populations [16]. During the mid-19^th^ century, entire boatfuls of fish were regularly caught [17] and many species of skates, rays and sharks were observed, including tope sharks (*Galeorhinus galeus*, Triakidae) and blue sharks (*Prionace glauca*, Carcharhinidae) [18]. Dogfish could be so numerous that fishers perceived them as serious pests. Their sharp spines entangled them in fishers' nets as they ate trapped fish [19], tearing and rendering the nets useless. Sometimes fishing would be brought to a halt until dogfish had moved out of an area [20].

**Table 1:** Timeline of notable events in Firth of Clyde fisheries.

Year	Action	Consequences for fishery
1838	Introduction of seine net	Increased numbers of herring caught, including juveniles
1860–67	Ballantrae Banks closed January–May	An attempt to prevent the capture of spawning herring
1867	All regulations on fishing in the open sea repealed as a result of the Royal Commission of 1863	‘Freedom of the seas’ ensues, previous legislation and byelaws repealed
1880s	Steam trawl vessels come into regular use	Steam power enables trawlers to expand fishing opportunities
1887	Fishery Board for Scotland conduct an enquiry into trawling	Enquiry leads to calls from some fishery scientists for the Firth of Clyde to be closed to trawling
1889	It becomes unlawful to trawl within three nautical miles of the low water mark all around Scotland. Whole Firth of Clyde closed to steam trawlers	Trawling (with the exception of sailing vessels less than eight tonnes) is stopped throughout the Firth of Clyde
1890s	Seine net becomes most common method of fishing for herring	Widespread use of seine nets allow greater catches to be taken
1920s	Some Clyde boats fitted with a motorised winch	Increases fishers ability to haul larger and heavier nets
1930s	Feeling wire put into use	Enables fishers to ‘feel’ for herring in the water column. Before this fishers had to locate herring shoals by sight alone
1950s	Introduction of echo sounders	Enables fishers to more easily detect concentrations of herring
1962	Autumn herring fishing season halted	Fishing season halted due to a lack of herring
1962	Byelaw introduced allowing trawling for *Nephrops* throughout Firth of Clyde (but not within 3 nm limit)	Most of the Clyde fleet switch to full-time demersal trawling
1960s	Mid-water trawl for herring comes into use	Allows fishers to continue fishing for herring even when shoals do not occur
1972	Seasonal closure implemented during spring herring fishery	Attempt to increase protection for dwindling herring stocks
1976	Quotas implemented in autumn herring fishery	Attempt to increase protection for dwindling herring stocks
1984	Three nautical mile trawl closure byelaw repealed	Allows fishers to expand their fishing opportunities during a time when demersal fin-fish landings are in decline

From the mid- to late-19^th^ century, the herring fishery in the Firth of Clyde was prosecuted mostly by small rowing and sailing skiffs [21]. Three main types of gear were used: the drift, trammel and seine net [9]. Drift nets were set behind the boat [21], while trammel nets were set upon the seabed to ensnare spawning herring [9]. Trammel nets were the major mode of herring fishing in the Firth of Clyde for centuries, but the seine net was adopted in 1838 in Loch Fyne (see Figure 1), and its use spread to other areas of the Firth of Clyde. It was known as the ‘ring-net’ (a precursor of today's purse seine), because fishers encircled the net around the shoal before drawing it tight to catch the fish.

The herring fishery was subject to fluctuations. Different areas around the coast would become productive, only to decrease a few years later. Loch Fyne in the inner Firth of Clyde was the site of a major spawning herring fishery in the mid-1800s, but by the 1880s few herring could be found there [14]. In 1895 the herring fishery was described as a failure in the Rothesay district in the Firth of Clyde [22], yet in 1897 a shoal several miles wide was seen [23].

**Figure 2:**
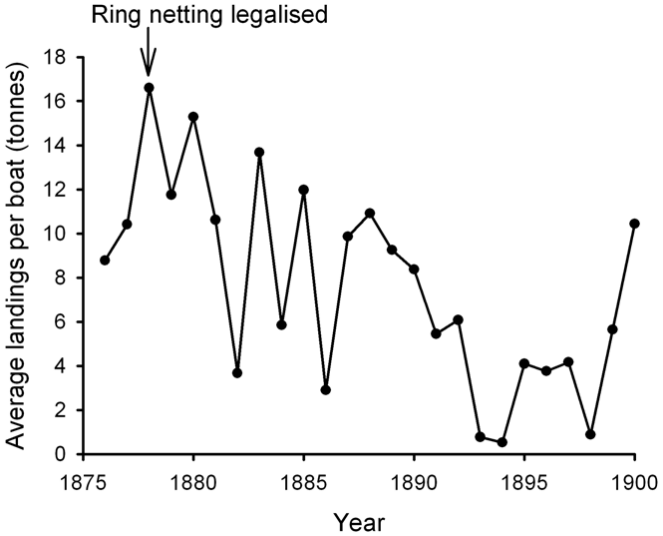
Average landings of herring per boat from Ballantrae Bank. Seines (ring-nets) were legalised in 1867 but were not put to use on the bank until 1878. These allowed much greater catches of herring to be taken, but over time average landings per boat declined. Source: Fulton (1900).

From 1860, the Ballantrae Banks were closed to fishing between January and May, as it was thought that the spawning fish were being caught before they could reach Loch Fyne, but this law was repealed in 1867 and the fishery resumed in 1868 [24]. However, conflict arose as it was claimed the large seine nets caused a great deal of destruction to deposited herring spawn upon the shallow banks. Although the Ballantrae grounds had long been subject to changes in fortunes of the herring fishery, Figure 2 shows that after the introduction of the seine net on the bank, average landings per boat declined. An enquiry into whether the seine net was injurious to drift net fishing was launched by the Fishery Board for Scotland in 1893 [24]. It concluded that the relatively shallow banks of the Ballantrae fishing ground led to the larger seine nets dragging along the sea bed where it damaged spawning habitat. This method of fishing was also more destructive to immature herring than trammel nets, as juvenile herrings were often unable to pass through the mesh [24] (see Appendix S1).

Despite concerns about the destructive effect of seine nets on the Ballantrae Banks, they were capable of taking hauls of over 200,000 fish [24]. The greater efficiency of the seine nets led to them becoming the most common method of fishing for herring in the Clyde by the 1890s [24]. By 1913, herring accounted for 84% of the total landings in the Clyde area, and 81% of the total value [25].

### A revolution for fisheries: the trawl

Most fishing for bottom-living species during the 19^th^ century was with static lines, nets and traps. However, the building of national rail networks brought swifter transport links to many coastal areas by mid-century. This meant that fish, a perishable commodity, could now be transported to inland towns and cities, creating an increased demand which encouraged more indiscriminate methods of fishing such as bottom trawling [26]. During the second half of the 19^th^ century, bottom trawls began to be used within the Firth of Clyde, dragged by small sailing vessels [27].

Sail trawlers relied upon favourable tides and winds to tow their gear along the seafloor, and were restricted to ground that was free from major obstructions such as boulders. However, just like the more efficient seine net for herrings, the trawl created conflict with other fishers as trawlers encroached upon traditional fishing grounds and created competition in the markets. Line fishers spoke of declines in inshore fishing grounds [28], yet the quantity brought in by trawlers increased as their gear and vessels became larger and more efficient.

A Royal Commission was established in 1863 in response to concerns about trawling and seine nets in fishing grounds, but none of the perceived problems were proved at this point, and the Commission even repealed all regulations upon fishing in the open sea, saying,

“Beam trawling in the open sea is not a wastefully destructive mode of fishing, but is one of the most copious and regular sources of the supply of eminently wholesome and nutritious fish. Any restriction upon this mode of fishing would be equivalent to a diminution of the supply of food to the people; while there is no reason to expect present or future benefit from that restriction” [29].

Further Royal Commissions in 1878 and 1885 failed to find evidence of declines in fish because of a lack of statistics [30]. In 1887, an enquiry by the Fishery Board of Scotland came to the conclusion that beam trawlers did damage other fishers' gear, and that immature fish were being killed in large numbers [29].

By the 1880s, the local sailing vessels worked almost everywhere in the lower reaches of the Clyde, where good catches of fish such as cod, dabs (*Limanda limanda*, Pleuronectidae), flounder, lemon soles (*Microstomus kitt*, Pleuronectidae) and turbot could be taken [27]. Yet to maintain a competitive edge, it was necessary that fishers continued to increase their efforts [14].

### The introduction of steam powered vessels

Despite concerns about trawling and its damaging effect upon fish populations and their habitat, the rapidly increasing human population continued to create a demand for cheap supplies of fish [16]. Whilst sail trawlers provided greater quantities and different species of fish than line vessels, they were still limited by tides and weather. However, during the 1880s a technological revolution occurred which was to change the face of fishing. Steam powered vessels came into regular use and rapidly replaced sailing vessels, transforming fishing for bottom-living species.

The introduction of steam gave trawling vessels an advantage in that they were no longer constrained by winds or tides, and could use larger and heavier gear. Chains could now be used as part of the trawl gear to tow over large obstructions, opening up grounds to fishing that sail trawlers had been unable to exploit [31]. Trawling therefore increased rapidly around the coast of Great Britain, and whilst fishing grounds further offshore began to be exploited, inshore areas still offered lucrative takes of fish. Trawling had always been viewed as an indiscriminate method of capturing fish, but the arrival of steam power vastly increased the fishing power of trawling vessels. The large quantities of undersized and non-commercial species thrown overboard and the perceived destructive nature of the trawl gear on fishing grounds led to further concerns among fishers of inshore areas such as the Firth of Clyde.

Some fishers interviewed as part of the 1887 enquiry had worked in the Clyde area for 40 years or more and witnessed the evolution of the trawler and seine net and consequent increases in discarding. They were also concerned about impacts of trawling on fish spawn, which they believed was deposited on the seabed [17] (see Appendix S1). Concerns about trawling impacts on spawn were justified in the case of bottom-spawning herring, but not in the case of most other commercially valuable species. A boat owner (A. Campbell), interviewed in 1887, voiced the opinion that,

“…if beam trawling is allowed to go on unchecked, the chief fishing banks in the Clyde (already greatly exhausted) will soon be so destroyed that for many years the yield will not meet the working expenses” [17].

However, the superior catching power of the beam trawl meant that fishing only increased.

### The closure of the Firth of Clyde to trawling

During the 1880s, and as a result of their enquiries, it became clear to a number of fisheries scientists that Firth of Clyde fisheries were becoming depleted (see Appendix S1). This led to calls for part of the Firth of Clyde to be closed to trawling:

“…From the scientific evidence obtained, and from the testimony given on the spot, it appears that the numbers of these fish have very seriously diminished in recent years; and it is scarcely possible to escape the conviction that this has been mainly due to excessive trawling […] There is reason to believe that were a period of quiescence bestowed upon some of these waters, opportunity would be given for undisturbed increase, especially of the smaller fish; and this would ultimately largely add to the yield, not only in the waters immediately protected, but in those which are contiguous” [32] (see Appendix S1).

**Figure 3:**
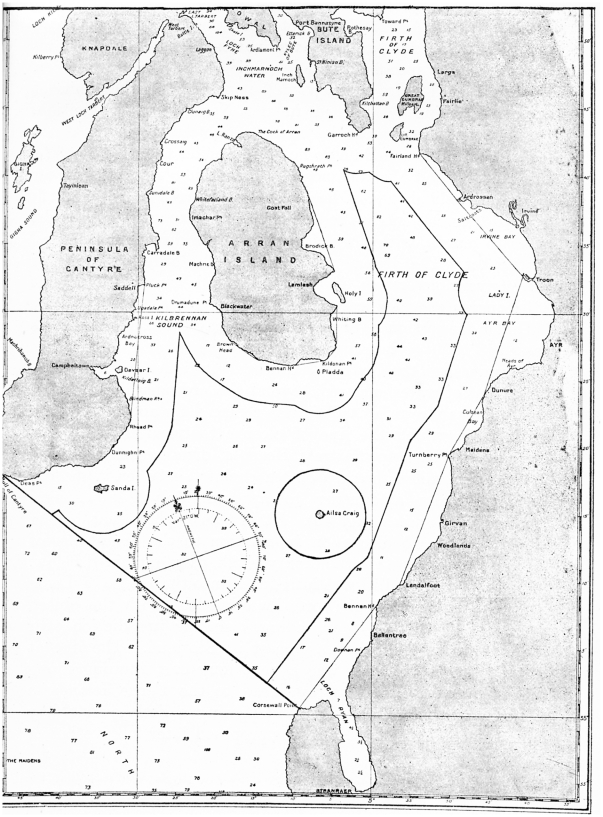
Areas closed to bottom trawling in the Firth of Clyde in 1889. The continuous line adjacent to the shore shows the limits of the three mile ban on trawlers greater than eight tons. The line across the outer Firth of Clyde encloses the area to the north from which steam trawlers were excluded. Effectively the whole of the Firth of Clyde was protected from 1889 to 1962 from large trawling vessels. Source: Fulton (1911).

These arguments against trawling were echoed throughout Scotland and, in 1889, it was made unlawful to trawl within three nautical miles of the low-water mark anywhere in Scotland. However, a bye-law was granted at the request of fishers from the Clyde district that allowed small sailing vessels less than eight tonnes to continue to trawl within the three-mile limit [33]. In the same year an area within the Firth of Clyde comprised of 380 square miles was also closed to bottom trawling, in a straight line from the Mull of Kintyre, Argyllshire, to Corsewall Point, Wigtownshire, and extending over part of the outer Firth of Clyde [34], [35] (Figure 3).

Although it had become clear by the 1880s that trawling would become the dominant method of catching whitefish [17], the expansion of and improvements in other fishing techniques, including line fishing, also caused declines in stocks. Professor J.C. Ewart and colleagues reported in 1888,

“The result appears to be a drain upon the supply of fish from the inshore waters sufficient, even without trawling, to cause diminution” [29].

Declines in fish stocks were also reported from other countries bordering the North Sea, with a similar pattern everywhere: a decrease in fish but improved catching technology as steam vessels became faster [36], and an expansion in the fish trade [37]. By the end of the 19^th^ century it was clear that coastal fish stocks were no longer inexhaustible [38].

### The increasing exploitation of Clyde fisheries

By the end of the 19^th^ century, steam had revolutionised the fishing industry. In 1895 the otter trawl was introduced in which, instead of a beam, the net was kept open by two boards that acted as hydroplanes. Otter trawls had a broader sweep and could capture fish much more efficiently than the beam trawl. Within one year of its introduction, the otter trawl had been adopted by almost all trawlers within the Scottish fleet [39].

Diesel motorboats also quickly increased in use before the onset of the First World War. The impact of motor power on the fishing industry was as considerable and almost as rapid as the invention of the otter trawl. By the first decade of the twentieth century, boats could travel further than ever offshore, with up to 240 miles travelled to fishing grounds (there were also exploratory forays to Arctic waters by east coast boats), whereas 40 miles was considered a long distance just a few years before [40]. This innovation also meant that motor-powered boats could be back hours before the sailing boats, saving valuable time [40].

In 1910 the Loch Fyne herring fishery was at its lowest ebb, and showed few signs of improvement from a state of depression that had become the longest on record [40]. Drift nets were gradually abandoned as more people turned to seine netting using motorised boats to catch herring [40]. However, by the 1920s the Loch Fyne fishery had started to revive, another example of the fluctuations common to herring [41]. Around this time, pressure also began to be placed upon opening up the closed area in the Clyde to trawlers [42] as foreign vessels increasingly frequented the boundaries of the closed area to fish [43].

The Clyde fishery saw new innovations in the mid-1920s, when two decked boats were built for Campbeltown fishers that were 50ft (15m) in length. Their catching power was further enhanced by the fitting of a motorised winch to haul in the net [42]. The rapid innovations in technology led to much more efficient vessels. By the end of the 1920s overall Scottish fisheries catches had more than doubled in a decade, from 156,460 tonnes in 1917 to 328,226 tonnes in 1926 [44], largely due to better technology and expanded fishing grounds.

However, inshore areas that had once been renowned for their large catches were suffering from more efficient fishing techniques. By the beginning of the 1930s, catches of herring in the Clyde had once again declined and fishers were now dependent on immature herring [45], [46].

Scientists established that the Clyde herring fishery was largely dependent on one year class at a time. Successful recruitment only occurred sporadically, accounting for the large year-to-year differences in catches [46]. Regulations were applied limiting the quantity of small herring that could be landed each day [47]; however this would have been unlikely to halt the destruction of juvenile fish as the fishing gear in use could not select just large fish. Therefore these regulations would simply have increased the amount of discarding.

Locating herring had for centuries been done by sight, using the presence of predators such as whales or gannets as clues, but in the 1930s ‘feeling wires’ came into use. These were weighted wires trailed from the deck of the fishing boat, from which skilled fishers would be able to locate the presence of herring as the wire brushed past their bodies [21]. Feeling wires were able to detect fish when the long-used techniques for sighting herring were unable to be employed because of unfavourable weather conditions, and were used until seine netting itself was discontinued in the 1970s [21].

With the onset of the Second World War many fishing grounds were protected from fishing due to hostilities, but within the Firth of Clyde most of the herring fishing areas remained available, with the inner waters of the Clyde opened when shoals of herring were suspected [48]. The importance of the Firth of Clyde area at this time was such that in 1940 it provided up to 43% of the total Scottish landings of herring [48].

### The re-opening of the Firth of Clyde to trawling

During the 1950s, the seine net fishery for herring was the most important and valuable in the Firth [19]. However, within a few years, herring landings were once again in decline, with catches consisting mainly of juvenile fish [49]. By 1962, there was such a lack of herring during the autumn that fishing was stopped for the season [50].

As the herring fishery declined pressure mounted to re-open the areas in the Clyde that had been closed to bottom trawling. It was argued that fishers needed to expand and diversify into other stocks, such as demersal fish species, scallops and *Nephrops*. A directed fishery for *Nephrops* began in the 1950s and quickly increased in importance in the Clyde area and the rest of Scotland. Seine nets were used in the Clyde until a Byelaw came into effect in 1962 which allowed trawling for *Nephrops* within the Clyde sea area that had been closed to trawlers since 1889, except within the three-mile limit [51]. The increase in *Nephrops* trawling also enhanced fish catches as any valuable species caught would be retained for market [52].

**Figure 4:**
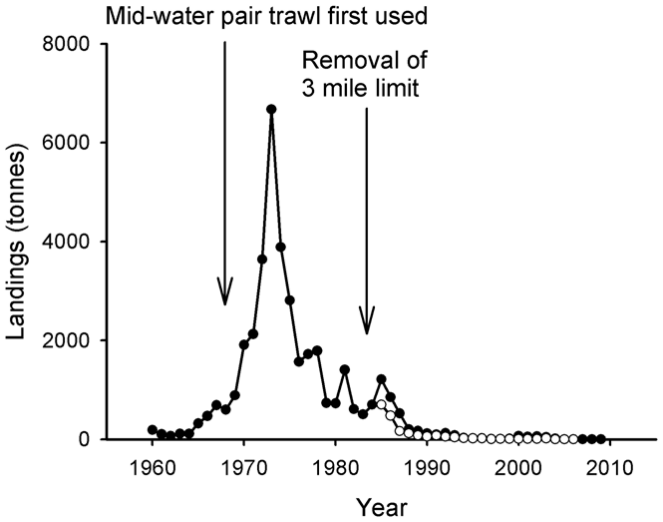
Landings of saithe. Closed circles indicate landings from the Wider Firth of Clyde, ICES statistical rectangles 39E4, 39E5, 40E4 and 40E5 1960–2009 (these encompass the Firth of Clyde, part of the North Channel and part of the Sound of Jura). Data sourced from Hislop (1986) (1960–1984), the Scottish Government (1985–1999) and Marine Fisheries Agency (2000–2009). Open circles indicate landings from the Inner Firth of Clyde zone (1985–2008). Data sourced from the Scottish Government (1985–2008).

The re-opening of the closed area to trawlers and the success of the demersal fisheries during the 1960s encouraged most of the Clyde fleet to switch to full-time demersal trawling [53]. As trawlers increased in power and adopted rock-hopper gear (rollers attached to the ground rope of the net) they began to operate in grounds that had previously been too rugged for fishing [53]. Catches of species such as cod and haddock swelled during the 1960s, whilst the invention of the faster mid-water pair trawl in the 1970s meant that large specimens of cod, hake (*Merluccius merluccius*, Merlucciidae) and saithe (*Pollachius virens*, Gadidae) were also caught in great quantities. Figure 4 shows a boom and bust in the saithe fishery that corresponds to the introduction of this gear which allowed mid-water shoals of the species to be caught with great efficiency.

Fishers who continued to target herring switched to pair-trawls during the late 1960s which rapidly became the predominant method [54]. With this invention, dense shoals of herring no longer had to be located before nets were set, as the mid-water trawl could be towed behind the boats to capture herring gradually. Towing along the mid-water column also allowed fishers to catch many of the larger, supposedly demersal fish, as the increased speed that nets were dragged meant that fewer animals could out-swim the boats [53].

**Figure 5:**
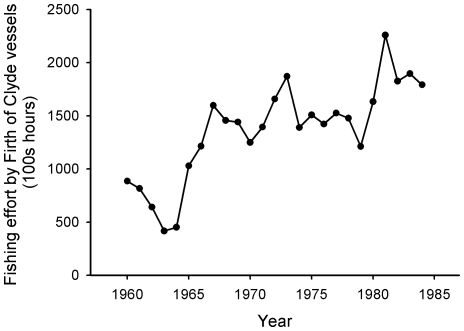
Fishing effort (100s hours) per year by vessels operating in the Firth of Clyde. Gear types included are *Nephrops* trawls, light trawl and seine nets. Despite the increase in fishing effort during the 1980s, landings of many bottomfish were already in decline. Data from Hislop (1986).

Fisheries for bottom-living species in the Clyde increased in importance as the herring fishery declined. However, the initial high landings during the 1960s and 1970s were not maintained. The three mile limit within which trawling had been banned since 1889 was repealed in 1984 to expand fishing opportunities as fishers struggled to maintain catches [55]. From then on the whole of the Firth of Clyde became accessible to trawling. As trawling expanded, exploitation of species such as cod, whiting, saithe and haddock intensified. Despite increased fishing effort (Figure 5) and enlarged fishing grounds, landings of bottomfish species could not be sustained and most stocks collapsed into the 1990s.

**Figure 6:**
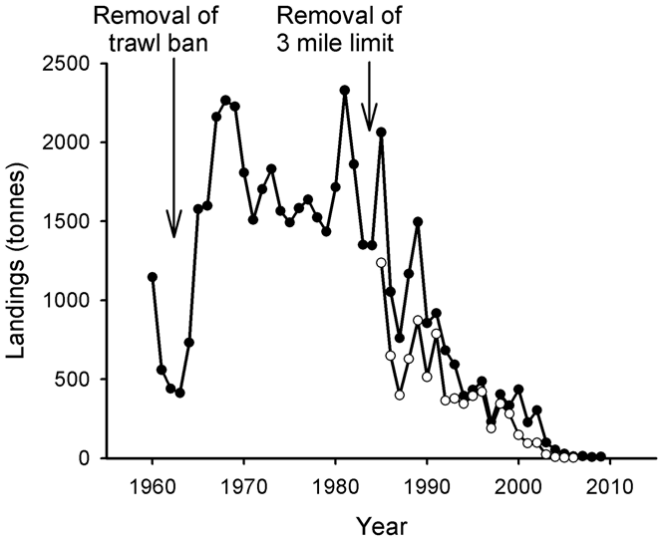
Landings of cod. Closed circles indicate landings from the Wider Firth of Clyde, and open circles landings from the Inner Firth of Clyde (see legend to Figure 4 for details).

Figure 6 shows that landings of cod increased rapidly during the 1960s following the reopening of the outer trawl closure area, remaining in a state of dynamic stability consistent with total effort until 1984 when declines began. However, the reopening of the three mile limit to trawling that year did little to raise landings, and between 1984 and 2009, landings decreased by over 99%. Despite recent measures such as minimum mesh sizes and temporary fishery closures to protect spawning cod stocks [56], cod spawning biomass around the Firth of Clyde has reached an historical low [57].

**Figure 7:**
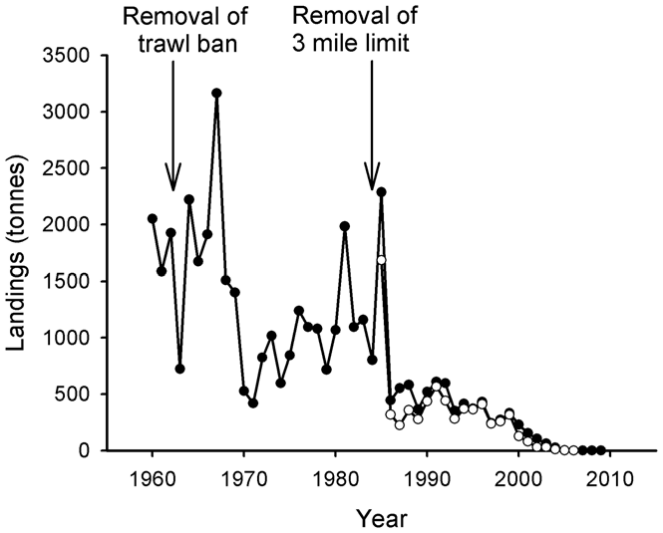
Landings of whiting. Closed circles indicate landings from the Wider Firth of Clyde, and open circles landings from the Inner Firth of Clyde (see legend to Figure 4 for details).

**Figure 8:**
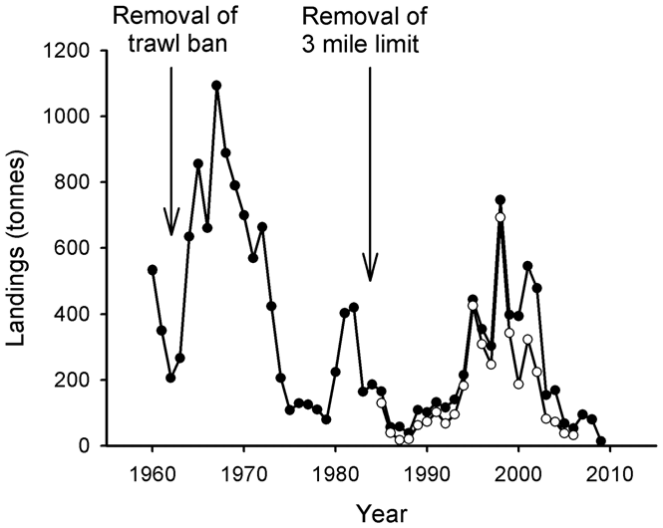
Landings of haddock. Closed circles indicate landings from the Wider Firth of Clyde, and open circles landings from the Inner Firth of Clyde (see legend to Figure 4 for details).

Landings of whiting (Figure 7) follow a similar trajectory of collapse to cod, with a decline of over 99% since 1984. Haddock (Figure 8) again follows a similar pattern, with short-term boosts in landings following the repeal of both trawl closures. However, landings have since collapsed and there has been an overall decline of 92% since 1984.

**Figure 9:**
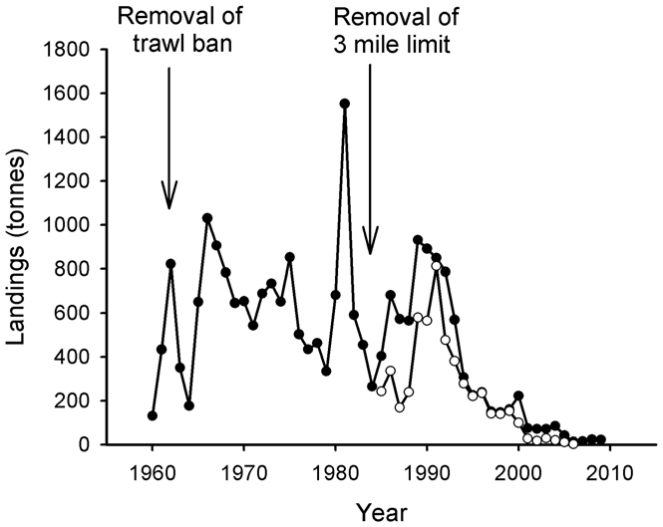
Landings of hake. Closed circles indicate landings from the Wider Firth of Clyde, and open circles landings from the Inner Firth of Clyde (see legend to Figure 4 for details).

Along with cod, hake (Figure 9) was one of the most important species in terms of value up to the 1980s, with up to 57% of the total Scottish landings of hake taken from the Clyde in the early 1980s [52]. Since the reopening of the trawl closure, landings have declined to virtually zero.

Landings of saithe peaked at over 6500 tonnes in 1973 following the introduction of mid-water trawls and accounted for over 50% of demersal landings in the Clyde that year [52]. In the mid-1970s higher prices were paid for all whitefish due to the establishment of Exclusive Economic Zones (EEZs) which closed off many traditional distant water fishing grounds to British fleets. The price of saithe was particularly high [52], encouraging greater landings. However, this was a boom-and-bust fishery and the catch quickly collapsed (Figure 4).

**Figure 10:**
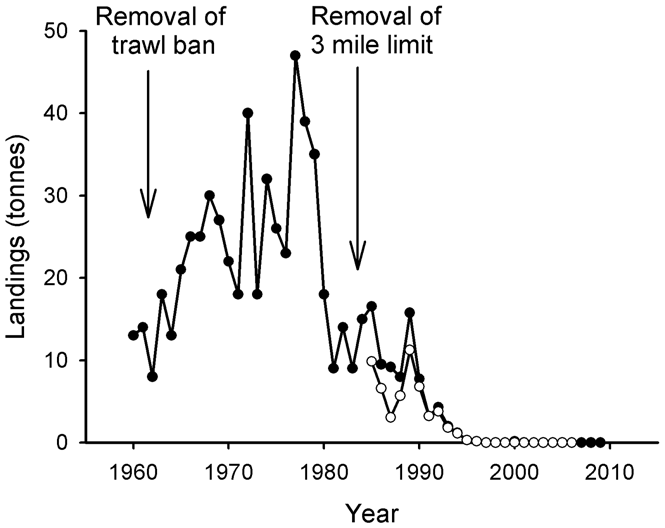
Landings of flounder. Closed circles indicate landings from the Wider Firth of Clyde, and open circles landings from the Inner Firth of Clyde (see legend to Figure 4 for details).

**Figure 11:**
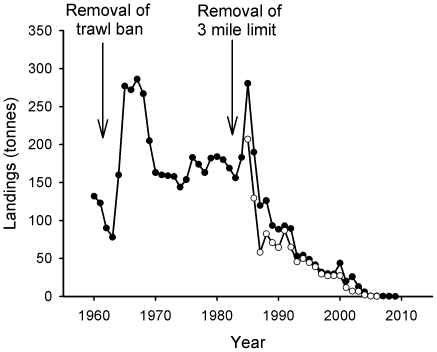
Landings of plaice. Closed circles indicate landings from the Wider Firth of Clyde, and open circles landings from the Inner Firth of Clyde (see legend to Figure 4 for details).

Flatfish landings such as flounder and plaice (*Pleuronectes platessa*, Pleuronectidae) have also declined (Figures 10 and 11). The low abundance of plaice in the Firth of Clyde was questioned more than a century ago, when a substantial difference in the number of flatfish between the Clyde and the Firth of Forth was noticed [58]. These investigations speeded the closure of the Clyde grounds to trawling, which, as noted earlier, lasted until 1962 and 1984 [51].

The early 1980s also marks the point when the Common Fisheries Policy was implemented and a number of key stocks began to be managed by Total Allowable Catch (TAC). Since 1984, TACs for species such as cod, haddock, whiting and plaice have decreased as stocks have declined. However, they have failed to promote recovery of demersal fish stocks [59]. Whilst some of the declines in landings may have been due to changes in market demand and alterations in the distribution of landings [60], for most of these species, they had simply been fished too intensively. This conclusion is supported by patterns in landings during the 1960s and '70s, as landings increased sharply after the reopening of the outer Firth of Clyde to trawling in 1962, but failed to respond for more than the first year to the reopening of the three mile closure in 1984 (Figure 3).

Landings data suggest that many bottomfish populations are now at an all time low, a view upheld by the personal testimonies of many experienced Clyde fishers [61].The conclusion seems inescapable that trawling closures provided important partial refuges for many commercially important whitefish species from the late 19^th^ century up until 1962 and 1984 when they were reopened. The protected effects of trawl closures were most likely achieved through a combination of habitat protection and reduced fishing pressure. The high fishing effort and damage to seabed habitats which immediately followed the re-opening of areas closed to trawling appears to have precipitated the complete collapse of the Clyde's demersal fin-fisheries.

**Figure 12:**
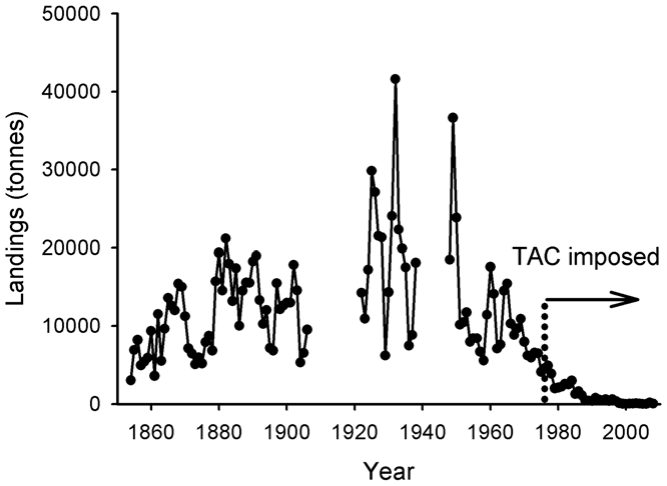
Landings of herring from the Firth of Clyde area from 1854 to 2008. Data from the Fishery Board for Scotland (1854–1937), the Department for Agriculture and Fisheries for Scotland (1938–1984) and the Scottish Government (1985–2008). In 1972 a Byelaw was introduced prohibiting all fishing for herring in the Firth of Clyde from 1^st^ January to 31^st^ March due to poor recruitment over a number of years. National quotas in the form of Total Allowable Catches (TACs) were set for the first time in 1976, and continue to the present day.

Figure 12 shows landings of herring from the Firth of Clyde districts from 1854 until 2008. The herring fishery, as previously described, has during its long history shown substantial variation in productivity, as is common in fisheries for pelagic species [62]. Nonetheless, it is possible to distinguish three phases in the fishery from the pattern of landings. The first is of development and industrialisation from the middle to late 19^th^ century, which corresponds to the expansion of the fleet and the introduction of steam drift netting boats. Then between the two World Wars the Firth of Clyde herring fishery saw an unrivalled prosperity, despite landings throughout the rest of Scotland waning during this period. The third phase occurs during the latter half of the 20^th^ century, when the Clyde herring fishery went into decline as technological innovations raised fishing pressure to unsustainable levels.

The Firth of Clyde herring fishery had until the 1970s been composed of two distinct fisheries. Almost the entire basis for the historical spring herring fishery in the Clyde were the spawning fish on Ballantrae Bank, which had been important since at least the 15^th^ century [54]. Herring would also be taken in areas such as Loch Fyne as fishers followed the migrating fish [54]. An autumn herring fishery was also prosecuted in the inner Firth of Clyde. However, by the late 1960s and early 1970s very few spring spawners arrived on the Bank and a seasonal closure was implemented from 1972 [51]. The autumn fishery was continued but to increase protection to the Clyde herring populations quotas were introduced from 1976. Today, quotas for herring in the Clyde are set at the low level of 1000 tonnes, reflecting just how badly this fishery has declined.

### Other impacts affecting the Clyde

The Firth of Clyde has been subject to a variety of human impacts alongside fishing activities. Throughout the 18^th^ and 19^th^ centuries, mill and manufacturing industries increased rapidly, leading to an influx of people to the nearby city of Glasgow and surrounding areas [63]. The upper Clyde estuary and associated rivers became grossly polluted from industrial and sewage effluents, reducing stocks of migratory fish such as salmon (*Salmo salar*, Salmonidae) and eels (*Anguilla Anguilla*, Anguillidae) [64]. Authorities attempted to improve matters during the late 19^th^ century by constructing several large sewage works [63]. However the Clyde remained heavily polluted and by the 1960s many of the rivers that drained into the Firth of Clyde were too polluted to hold fish populations [65]. Legislative measures were introduced from the 1950s to reduce pollution levels, which began to take effect during the 1970s [63]. Pollution doubtless put pressure on species inhabiting the area, but the timing of fish population declines in relation to developments in the fishery make it clear that it was fishing, not other human impacts, that has primary responsibility for the downfall of Clyde fish populations.

### Transformation of the Firth of Clyde

**Figure 13:**
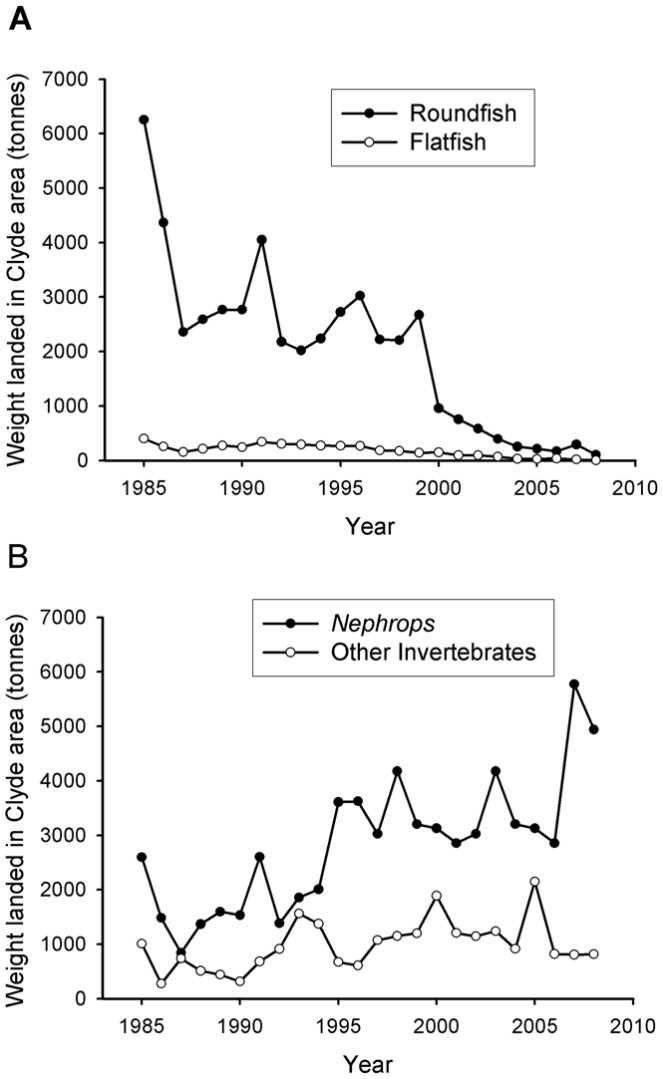
Landings of species' groups from the Firth of Clyde from 1985 to 2008. Overall landings peaked at over 10,000 tonnes in 1985 but have since decreased. (a) Flatfish and roundfish landings have declined to almost zero. (b) Invertebrate landings have increased with *Nephrops* now dominating catches. Data from the Scottish Government.

**Figure 14:**
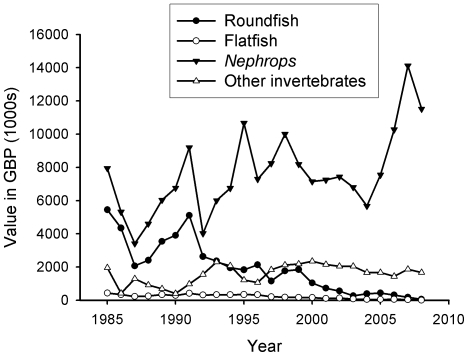
Value of landings into the Firth of Clyde from 1985 to 2008. *Nephrops* now dominate landings by value (values corrected for inflation using www.thisismoney.co.uk/historic-inflation-calculator). Data from the Scottish Government.

In 2008, finfish such as cod, plaice and herring constituted less than 2% of landings by weight (Figure 13a). Eighty-four percent of landings by weight were *Nephrops* (Figure 13b), representing nearly 87% of the value of Clyde fisheries (Figure 14). The remainder was composed of other invertebrates such as scallops, crabs and lobsters.

*Nephrops* is a small crustacean that inhabits burrows in fine muddy sediment. It is predated by finfish species such as cod [66] and today is one of the most commercially important fisheries in the Northeast Atlantic. Total demersal catch of *Nephrops* increased in the 1960s and 1970s due to a rise in trawling for this species [52], which came about as prices increased and as fishers diversified because of dwindling fish stocks. Falling populations of demersal fish likely boosted stocks of *Nephrops* as their predators declined. *Nephrops* had become the most valuable species landed in Scotland by the 1970s, with the 1972 total Scottish catch valued at £3.9m [67]. *Nephrops* is now the most valuable fishery in the Clyde, targeted by around 120 vessels, of which 90% of landings are made by resident Clyde trawlers [56].

The stock is currently considered as being sustainably fished [68], and restrictions on mesh size and days at sea are in place to prevent over-exploitation [69], but these measures do nothing to protect benthic habitats from the damage caused by this form of fishing or removal of non-target species, especially juveniles of fish such as cod, haddock and plaice. Discard ratios are very high in the Clyde *Nephrops* fishery, with 9kg of bycatch produced for every 1kg of *Nephrops* caught [69]. 25,000 tonnes of discards are generated every year in the Firth of Clyde from *Nephrops* trawling alone [70], and it is likely that many of these organisms die when returned to the sea [71]. The other fishery that remains is for scallops which are caught using heavy steel dredges with teeth that tear up the sea bottom. This method of fishing has been shown to destroy biologically sensitive habitats and alter marine ecosystem functioning [72], and will therefore exacerbate impacts of *Nephrops* trawling.

**Figure 15:**
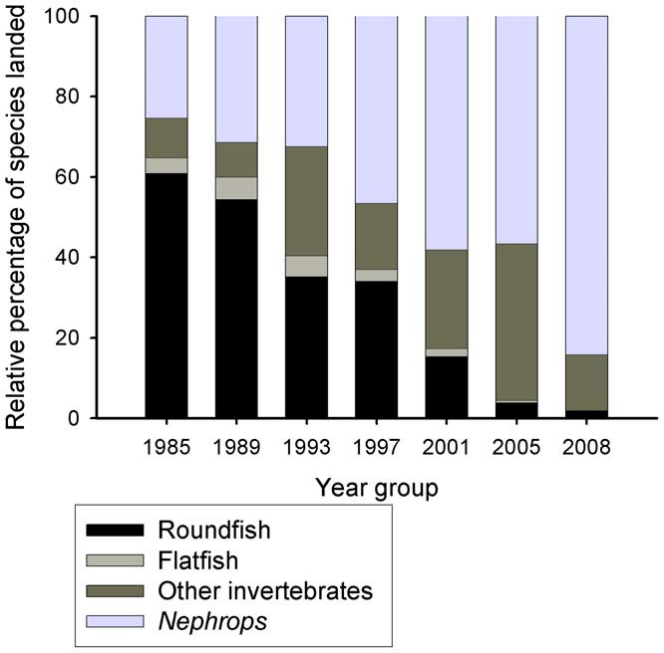
Relative proportions of species landed in the Clyde from 1985 to 2008. *Nephrops* now dominate landings, and landings of other invertebrates have increased. The proportion of finfish has declined from nearly two-thirds of landings in 1985 to just 2% in 2008. Data from the Scottish Government.

Over the course of two decades there has been a remarkable shift in the groups of species landed from the Clyde. In 1985, finfish made up over 60% of the landings by weight and 37% by value, compared to just 2% by weight and 0.5% by value in 2008 (Figure 15). Towards the end of the 20^th^ century, species landed at Clyde ports shifted from predatory fish to invertebrate species of a lower trophic level. *Nephrops* landings into Clyde ports have plateaued in recent years (Figure 13b).

## Discussion

It is clear from this compilation of long-term data and personal testimonies from those associated with the Firth of Clyde, today [61] and in the past, that many once abundant species are now ecologically extinct in the Firth of Clyde ecosystem. Throughout the documented history of the herring fishery, large fluctuations had occurred in stocks and catches. At the turn of the 20^th^ century, spawning herring numbers on the Ballantrae Banks had collapsed, yet by 1958 the Banks had seen a resurgence with an estimated 50 million spawning herring present that year [73]. But over time, the amount of juvenile herring taken became too great; seine nets were capable of capturing herring long before they reached maturity [74], whilst mid-water trawls could be used effectively when large shoals did not occur [53]. These intensive fishing methods, coupled with weak recruitment, led to the 1977 Scottish herring landings being the lowest since the beginning of the 20^th^ century [75]. Seasonal closures and quotas were implemented during the 1970s, but the recovery of herring was slower than expected [76], and has so far failed to occur in the Firth of Clyde.

The collapse of bottomfish fisheries followed closely upon the removal of the three nautical mile closure to bottom trawling in 1984. Although there is no direct evidence to link the removal of this refuge to the fisheries collapse, recent research on areas protected from mobile fishing gears in the US Gulf of Maine [77], [78], Emerald Bank Canada [79] and Icelandic continental shelf [80] have shown strong positive effects of such closures on spawning stock biomass of several species including haddock, flounders, cod and scallops. It seems likely that protection from bottom trawling in the Firth of Clyde had similar benefits to fish stock protection and helped sustain landings of bottomfish until the 1980s. Trawl closures had no such beneficial effect on pelagic herring fisheries, which collapsed in the late 20^th^ century, largely due to the introduction of highly efficient mid-water trawling and fish finding technology.

Recent diving expeditions have shown there to be limited patches of seagrass (*Zostera* spp. Zosteraceae) present in the Firth of Clyde [81], [82], however there is little information regarding the historical extent of these habitats. It is likely that seagrass beds within the Firth of Clyde declined as a result of direct impacts such as dredging, trawling and construction, indirect pollution effects and disease [83]. Seagrass beds are known to provide important spawning and feeding areas for a variety of species, including fish species such as cod and herring [84]. Hence it is possible that declines in seagrass habitat had a negative effect upon commercial fish populations, especially due to the fall off in suitable habitat in which herring could lay their eggs.

As well as removing bottomfish species, bottom trawling and scallop dredging have reduced habitat complexity in the Clyde resulting in an environment that supports low macrofaunal diversity [7], making it less resilient to environmental fluctuations [85]. In this altered ecosystem, *Nephrops* and scallops thrive as these species are adapted to the simplified environmental conditions and are now subject to much reduced predation pressure. Myers and Worm [86] investigated the effects of long-term exploitation on a variety of marine communities. They found that industrialised fisheries typically reduced unexploited community biomass by 80% in 15 years, whilst predatory fish biomass was estimated to be only around 10% of pre-industrial levels. More recent data and evidence from the Firth of Clyde suggest that losses of fish biomass over timescales of centuries have been greater, exceeding 95 or even 99% [31]. Frank et al. [87] found that the removal of top predators such as cod in a north-western Atlantic ecosystem resulted in a completely restructured food web, with implications for species at all trophic levels. Trophic cascades such as these can often result in lower trophic level species dominating the environment [88], which has occurred in the Firth of Clyde.

In combination, the habitat altering properties of *Nephrops* and scallop fisheries and high bycatch of juvenile fish associated with *Nephrops* trawls will prevent the recovery of bottomfish populations. In our view, the complexity and productivity of the Firth of Clyde ecosystem will only be restored with the (re)introduction of significant spatial protection from fishing. Areas must at least be closed to mobile fishing gears [89], but greater benefits to a wider variety of species, both targets of fishing and non-target species, would come from full protection from exploitation in a network of marine reserves [90], [91].

Although trawl fisheries for *Nephrops* are currently sustaining catches, creating the appearance of sustainability, studies have shown that populations in low diversity ecosystems are naturally less stable than those in more diverse ones as they are at higher risk from disease, invasion and changes in environmental conditions [92]. This makes current fishing practise in the Clyde highly risky. There are already signs of high rates of parasitism of Clyde *Nephrops* by the dinoflagellate *Haematodinium*, reaching as much as 70% infection rates [93]. This parasite renders the prawns valueless. The parasite impairs swimming ability of *Nephrops* and infected prawns spend more time out of their burrows [94]. In the past, parasitized prawns would have been picked off by predators, but in the reduced predation environment of the present day Firth of Clyde, levels of parasitism have increased. In terrestrial settings, adverse effects of ecosystem simplification in agriculture (weeds, pests and diseases) are combated by application of chemicals. Such a remedy is not practical or desirable in the sea as the costs would be prohibitive and the application of chemicals cannot be controlled so easily. It is important, therefore, if fisheries production is to be sustained over the long-term, that management measures are put in place that protect the variety and abundance of life in the sea, as well as maintaining the physical and ecological complexity of marine ecosystems. It is deeply ironic that the Marine Stewardship Council certificate of sustainability has recently been awarded to another *Nephrops* fishery off the west coast of Scotland [95], a last-resort fishery that has replaced a once diverse mixed species fishery such as the Firth of Clyde had. At the time of writing, the Clyde *Nephrops* trawl fishery was under assessment.

Moves toward protection are being made. In September 2008, and after thirteen years of campaigning by the Community of Arran Seabed Trust (COAST), the Scottish Government created Scotland's first no-take zone in Lamlash Bay within the Firth of Clyde [96]. Whilst this is an important step forward, the area of no-take is just 2.67 square kilometres and will not be able to restore fisheries within the Firth of Clyde alone.

Fishing is not the only human impact to have affected the Firth of Clyde. As we noted above, this water body has been subject to high inputs of pollution from the City of Glasgow, as well as agricultural runoff, military and port developments, munitions and sewage dumping among others. Despite these problems, there is little evidence to suggest that pollution had any major effect on fish stocks in the wider Firth of Clyde. However, in the upper estuary it was found that once pollution levels began to decline, numbers of fish species increased [97], and in 1983, salmon returned to the river Clyde after an absence of nearly 150 years [98]. Whilst rivers and the inner estuary of the Clyde clearly suffered adversely from high levels of pollution [99], there is little evidence to support the role of pollution in declining marine stocks in the wider Firth of Clyde. If pollution effects were the cause of fish stock decline throughout the wider Firth of Clyde, fish landings would be expected to rise from the 1980s, rather than continue to decline. It is clear from our analysis that fishing has been *the* major driving force in its transformation.

The Clyde used to contain a great diversity and abundance of species, as was apparent from people's testimonies from before the advent of steam trawling. Nevertheless, just as steam trawling was introduced, the Firth of Clyde was already classed as ‘exhausted’ by leading 19^th^ century fisheries scientists [29]. Yet even the ‘degraded’ ecosystem they knew is no more, as trawling and technological improvements, as well as political decisions to open up closed areas have left fish nowhere to hide. Long-term landings trends reveal large shifts and declines in marine biodiversity. However, similar trends are being repeated all over the world as global biodiversity loss is increasing [5], and some marine species that were once assumed to be widespread, abundant and inexhaustible are in danger of extinction [100].

Fishery management in the Firth of Clyde and other similarly affected marine ecosystems needs to change from the current species-centred approach to one which takes into account the complex ecosystems that are present [92], [101]. Long before the current fishery problems in the Clyde emerged, it was clear to late 19^th^ century fisheries scientists that the Clyde ecosystem needed protection from overexploitation and damage by fishing gear [24], [32]. Their advice is even more relevant today as the Firth of Clyde approaches the endpoint of overfishing, the point where nothing remains that is worth catching. The region now faces possible irreversible losses of biodiversity, fisheries productivity and other important ecosystem services provided by species whose ecological roles have disappeared as their populations have collapsed.

The story of the Firth of Clyde is emblematic of wide experience in world fisheries. It illustrates how opportunity and necessity have driven fisheries expansion and innovation, first to increase catches, then to sustain them even as fish populations fell. Such adaptability and ingenuity in fishing cultures is often admired, yet the hard fact is that it is ultimately self-defeating, since fisheries can be exhausted. There can be few better examples than the Clyde of a place where fish stocks have been effectively mined out of existence, and fisheries management has so signally failed to prevent it. Nonetheless, the positive role that bottom trawl closures appear to have played in sustaining intensive fisheries throughout the mid-20^th^ century suggests that they, together with highly protected marine reserves, could help reverse the course of this particular, sad history, and other similar cases.

## Materials and Methods

We use Government landings data, alongside qualitative and anecdotal information from a variety of sources to describe changes in fisheries and the environment of the Firth of Clyde from the second half of the 19^th^ century to the present day. Historical information on herring landings were obtained from the Annual Reports of the Fishery Board for Scotland (1882–1939), Scottish Home Department reports (1948–1958), Department of Agriculture and Fisheries for Scotland (1959–1984) and the Scottish Government (1985–2008). Landings data for demersal fish species come from Hislop (1986) (1960–1984), the Scottish Government (1985–1999) and the Marine Fisheries Agency (2000–2009). The Scottish Government and Marine Fisheries Agency were contacted directly for data. These data show landings from ICES statistical rectangles 39E4, 39E5, 40E4 and 40E5 which encompass the Firth of Clyde, part of the North Channel and Sound of Jura [see 52]. We call this area the Wider Firth of Clyde. Shorter time series which show landings from the inner Firth of Clyde area only are available from 1985 (Scottish Government data). We refer to these data in figures as the Inner Firth of Clyde.

We recognise that landings data cannot directly reflect the status of fish populations within the Clyde and they do have limitations, as changes to landings distribution occurs over time. However, we have used a variety of additional sources to document changes to the Firth of Clyde over time that complement and underpin landings data to build a picture of an environment undergoing ecological meltdown.

## Supporting Information

Appendix S1 Descriptive extracts describing Firth of Clyde fisheries.
(0.04 MB DOC)
