# Supplementary material for: Ecological Meltdown in the Firth of Clyde, Scotland: Two Centuries of Change in a Coastal Marine Ecosystem
Source: PLoS One. 2010 Jul 29;5(7):e11767. doi: 10.1371/journal.pone.0011767 (PMC2912323; doi:10.1371/journal.pone.0011767)
Supplement: Appendix S1 — Descriptive extracts describing Firth of Clyde fisheries. (0.04 MB DOC) [file pone.0011767.s001.doc]

**Appendix S1: Descriptive extracts describing Firth of Clyde fisheries**

*Whitefish fisheries*

The following extract comes from H. Beaufoy in an account to the House of Commons in 1785, after he spoke to fishers who worked in the outer Firth of Clyde,

“One of these East country fishers was engaged by Mr. Campbell, of Islay, to remain with him. On conversing with him, he gave me accounts of the quantities of fish he catched, that appeared altogether incredible; for one article, he assured me, that when he baited his small line for the smaller flat fish, which line contains 400 hooks, it was not at all an uncommon thing for him to take in, at one haul, 350 fish from the 400 hooks. These consist of turbot, soal [sole], and large fine flounders, about two or three pounds weight each. As to skait [skate], he said he seldom fished for them, as they are not a saleable article there; but he could easily fill his boat with them, when he chose it, at one haul of his lines – and from the accounts of other people, I had no reason to doubt the man’s veracity” [[[1]](#endnote-2)].

Whilst descriptions such as these may seem unbelievable today, the author of the report made further enquiries with other people from the region, and came away satisfied that they were not exaggerating.

*Herring fisheries*

19^th^ century accounts describe the abundance and diversity of predators within the Clyde, many of which gathered to feed on the herring. For example as P. Wilson described in 1887,

“The banks are at times the scene of lively interest when visited by a shoal of whales. On a recent occasion, as the sun was setting, a shoal of at least forty whales in pairs and a number of porpoises began to play, and went circling round the margin of the bank displaying their huge fins and arched backs, gracefully plunging and again reappearing a short distance off. The porpoises, in wild leaps went several feet sheer out of the water, and then dived apparently in search of their prey. In this manner the flock of whales and porpoises went circling round for at least a distance of ten miles” [[[2]](#endnote-3)].

Wilson also describes the many seabirds attracted by the enormous herring shoals of the Ballantrae Banks,

“Overhead the air is often clouded with them [gannets]. Gathering their wings to their sides, they drop from the height of 200 to 300 yards like meteor showers, jets of spray rising on the surface, and indicating the spots where they have pierced the water. Sometimes they descend to a depth of several fathoms, and fish the herrings from the trammel-nets at the sea bottom, and occasionally get caught themselves in the meshes […]. All authorities agree in the opinion that the quantity of herrings consumed by the gannets exceeds that captured by man” [Error: Reference source not found].

The number of predators visible in the water and their ferocity when consuming the shoals of herring, left people amazed that there would be anything at the end of the frenzy for the fishers, as described by Wilson,

“With so many agencies of destruction, the marvel is that so many herrings remain to become the food of man, who, with all his ingenuity and skill, cannot compete with the sea birds, cod, and other fishes” [Error: Reference source not found].

Seine net fishing was more destructive to immature herring than traditional trammel nets, as T.W. Fulton described in 1900,

“With regard to the capture of small or immature herrings, there is no doubt that the seine net takes far greater quantities than the trammel net does […]. In seasons when small herrings abound very large quantities have been landed, and in some years have been found unsaleable and used for manure. Sometimes the catch of the seine boats consists of herrings as large and fine as those got by the trammels, but as a rule there is a considerable admixture of inferior fish. If the net is torn on the bottom, which is not an infrequent occurrence, or if a larger quantity is enclosed than the boats can well carry, and part is allowed to escape, the proportion of small herrings in the catch may be increased, inasmuch as large numbers of them are meshed” [[[3]](#endnote-4)].

*Royal Commissions*

As a result of widespread concerns about the impacts of bottom trawling and perceived declines in inshore areas, a Royal Commission of Enquiry was established in 1863. They concluded that beam trawling was not destructive, and recommended that fishing restrictions be lifted [[[4]](#endnote-5)]. However, concerns of overfishing in inshore waters continued,

“It is everywhere alleged that the territorial waters have been overfished, but in the absence of statistics it is impossible in the meantime to arrive at any definite conclusion on the subject. That there is no general exhaustion of the inshore and offshore waters around the coast may be inferred from the fact that nearly double the quantity of round fish (exclusive of herring) was landed in 1887 than in 1882, and that at Stonehaven and other centres, where trawling is not prohibited by the bye-laws, more fish were landed in 1887 than in former years […]. It may afterwards be shown, however, that inshore grounds can be exhausted by either line or beam trawl fishermen, the rate and extent depending on the number and size of boats fishing” [Error: Reference source not found].

*Perceived impacts of trawling*

Many fishers and others associated with the fishing industry viewed trawling as destructive. J. Wason, a postmaster from Ballantrae, interviewed in 1887 stated,

“Some fishermen present had seen the small fish shovelled overboard, and immense quantities of spawn also shovelled overboard” [[[5]](#endnote-6)].

W. Hannah had been a Firth of Clyde fisher for 40 years by 1887, when he was questioned about beam trawling. In 1888, Maitland and Ewart [Error: Reference source not foundError: Reference source not found] describe the discarding practises he had seen, as well as the decrease in abundances of fish,

“He had witnessed [a] trawl being hauled, the catch consisting of dog-fish, skate, &c., and a great amount of small fish, rowds (gurnots), turbot, flounders, and others. There were more small than big fish, and they were usually thrown overboard”.

“The supply of fish has been diminishing during the last 10 or 12 years, only 10 turbot being got now instead of from 40 to 80 got 15 years ago” [Error: Reference source not found].

A District Fishery Officer from Greenock also made his views clear regarding the destructiveness of beam trawling,

“At the tail of the [Ballantrae] bank, in particular, the takes have fallen off very much of late, and the fish now got are generally very much smaller in size than formerly, and altogether the aspect of things appear to indicate that beam trawling has injured this [whitefish] fishery very considerably” [Error: Reference source not found].

Yet to maintain a competitive edge, it was necessary that fishers continued to increase their efforts. G. Brooke wrote in 1886:

“It appears that so long as one man fishes in the day-time all must follow, if only in self-defence” [[[6]](#endnote-7)].

This led to calls from scientists for part of the Firth of Clyde to be closed to trawling, in line with recent closures in some east coast inshore areas.

“…In consequence of the state of matters thus revealed, it is extremely desirable to proceed to regulate as soon as possible beam trawling in the Clyde area, partly by way of protecting the spawning grounds, and partly in order to prevent the complete destruction of flat-fish. To admit of any real good being done, it will be necessary of the Board to have complete control over all the Firth, and not merely the upper reaches of the Clyde [...]. It is a noteworthy circumstance that recently an increase has occurred in the number of the smaller fish landed by line fishermen on the East Coast, especially from inshore waters, and this may be considered as an indication of more favourable conditions for natural reproduction” [[[7]](#endnote-8)].

1. . Beaufoy, H (1785) Third report from the Committee appointed to inquire into the state of the British fisheries, and into the most effectual means for their improvement and extension. Sixteenth Parliament of Great Britain: Second session (25 January 1785 - 2 August 1785). In: Reports from the Committees of the House of Commons 1715-1801 (1803). Miscellaneous Subjects Vol. 10: 1785-1801. pp. 18-189. [↑](#endnote-ref-2)
2. . Wilson P (1887) The herring fishing in Ballantrae district. In: Boyd TJ, Smith JG, Thoms GHM, Irvine AF, Maitland JRG et al., editors. Fifth annual report of the Fishery Board for Scotland, being for the year 1886. Edinburgh: HMSO. Part 1, Appendix J. pp. 431-434. [↑](#endnote-ref-3)
3. . Fulton TW (1900) Report of an enquiry on the action of the herring seine-net. In: Sutherland A, Crawford D, Thompson DW, Welch JR, Duguid WR et al., editors. Eighteenth annual report of the Fishery Board for Scotland, being for the year 1899. Edinburgh: HMSO. Part 3. pp. 242-271. [↑](#endnote-ref-4)
4. . Ewart JC, Maitland JRG, Boyd W, Johnston J (1888) Fishery Board for Scotland: Report of a committee of the Fishery Board for Scotland as to the regulation of trawling and other modes of fishing in the Territorial Waters. Edinburgh: HMSO. 120 p. [↑](#endnote-ref-5)
5. . Maitland JRG, Ewart JC (1888) Preliminary report and evidence taken by Sir James Maitland and Professor Ewart as to the influence of trawling and other modes of fishing in the Clyde estuary. In: Ewart JC, Maitland JRG, Boyd W, Johnston J, editors. Fishery Board for Scotland: report of a committee of the Fishery Board for Scotland as to the regulation of trawling and other modes of fishing in the Territorial Waters. Edinburgh: HMSO. Appendix A. pp. 3-11. [↑](#endnote-ref-6)
6. . Brook G (1886) Report on the herring fishery of Loch Fyne and the adjacent districts during 1885. In: Boyd TJ, Smith JG, Thoms GHM, Irvine AF, Maitland JRG et al., editors. Fourth annual report of the Fishery Board for Scotland, being for the year 1885. Edinburgh: HMSO. Part 1, Appendix F. pp. 47-60. [↑](#endnote-ref-7)
7. . Ewart JC, Maitland JRG (1888) Report on the trawling experiments of the Garland and field statistics of the East Coast fisheries. In: Boyd TJ, Smith JG, Thoms GHM, Irvine AF, Maitland JRG et al., editors. Sixth annual report of the Fishery Board for Scotland, being for the year 1887. Edinburgh: HMSO. Part 3. pp. 25-188. [↑](#endnote-ref-8)
